# Cell-type heterogeneity: Why we should adjust for it in epigenome and biomarker studies

**DOI:** 10.1186/s13148-022-01253-3

**Published:** 2022-02-28

**Authors:** Luo Qi, Andrew E. Teschendorff

**Affiliations:** 1 CAS Key Laboratory of Computational Biology, Shanghai Institute of Nutrition and Health, University of Chinese Academy of Sciences, Chinese Academy of Sciences, Shanghai, 200031 China; 2 UCL Cancer Institute, University College London, London, WC1E 8BT UK

**Keywords:** Cell-type heterogeneity, Cell-type deconvolution, Epigenetic biomarkers, DNA methylation, Classification

## Abstract

Most studies aiming to identify epigenetic biomarkers do so from complex tissues that are composed of many different cell-types. By definition, these cell-types vary substantially in terms of their epigenetic profiles. This cell-type specific variation among healthy cells is completely independent of the variation associated with disease, yet it dominates the epigenetic variability landscape. While cell-type composition of tissues can change in disease and this may provide accurate and reproducible biomarkers, not adjusting for the underlying cell-type heterogeneity may seriously limit the sensitivity and precision to detect disease-relevant biomarkers or hamper our understanding of such biomarkers. Given that computational and experimental tools for tackling cell-type heterogeneity are available, we here stress that future epigenetic biomarker studies should aim to provide estimates of underlying cell-type fractions for all samples in the study, and to identify biomarkers before and after adjustment for cell-type heterogeneity, in order to obtain a more complete and unbiased picture of the biomarker-landscape. This is critical, not only to improve reproducibility and for the eventual clinical application of such biomarkers, but importantly, to also improve our molecular understanding of disease itself.

## Background

The cell-types that are present within a given tissue or organ are distinguished by a unique gene and protein expression profile [1]. This functional molecular profile is epigenetically determined via a complex interplay of histone modifications, chromatin accessibility and covalent DNA methylation marks [2]. Thus, epigenetic profiles are highly cell-type specific.

Clinical interest in measuring epigenetic profiles stems from the fact that such epigenetic profiles are often altered in disease and in association with disease risk factors [3–9], with some evidence also pointing to a potentially causal or causally-mediating role [3, 10–12]. DNA-based epigenetic marks like DNA methylation (DNAm) are also fairly stable and amenable to genome-wide measurement in large numbers of samples and in many types of clinical specimens [13, 14], including blood [15–17], cell-free DNA in serum [18, 19] and formalin-fixed paraffin embedded (FFPE) tissue [20–22], making it a very attractive substrate for biomarker studies. Indeed, the sensitivity of technologies like the Illumina DNAm beadarray is such that one can detect DNAm changes as small as 1–5% with over 90% sensitivity [23, 24]. This high sensitivity and precision has been confirmed by many biomarker studies: for instance, smoking-associated DNAm changes in blood tissue are characterized by such small effect sizes and are highly reproducible [25, 26].

However, a key challenge for the biological interpretation of such epigenetic changes remains in that epigenetic measurements are generally performed on DNA extracted from heterogeneous sample specimens. This is because measuring epigenetic profiles, including DNA methylation, at cell-type resolution and for all cell-types in the tissue is currently very costly or technically challenging [27], and in the case of single-cells only generates very sparse and incomplete data [28, 29], which is therefore impractical for biomarker studies which aim to measure genome-wide profiles in hundreds if not thousands of clinical samples. Thus, the obtained measurements on bulk samples only reflects an average DNAm profile over all cells and cell-types within the specimen. It follows that this average DNAm profile will be affected by factors such as the cell-type composition of the sample, which could vary substantially between individuals. Thus, it might be difficult to ascertain in which cell-types a particular 5% change in DNAm is happening. Although similar considerations apply to other epigenetic marks such as histone modifications and chromatin accessibility, for reasons given above this perspective focuses on DNAm.

One of the first studies to demonstrate the big effect that cell-type heterogeneity (CTH) can have on subsequent statistical inference was an Epigenome-Wide Association Study (EWAS) performed in blood tissue from Rheumatoid Arthritis (RA) cases and controls [15]. In this study it was shown that there were a large number of CpGs differentially methylated between RA cases and controls, owing to a substantial shift in the granulocyte to lymphocyte proportions between cases and controls. While such alterations in blood composition could potentially be useful as a diagnostic marker (assuming they are specific to the disease), they don’t reflect disease-associated DNAm alterations that occur in a given cell-type. It is now widely recognized that the large number of differentially methylated cytosines (DMCs) detected in this RA EWAS study is a reflection of the inflammatory response to the disease, which is therefore of limited interest for identifying disease risk markers. As shown by Liu et al. the great majority of these DMCs disappear once we adjust for the underlying changes in cell-type composition between RA cases and controls [15]. Another important and more recent application where adjustment for CTH is critical, is in the construction of diagnostic and pre-diagnostic disease predictors (e.g. cancer) from cell-free DNAm in serum, where such adjustment is necessary to remove the contaminating effect of lymphocyte DNA [18, 19, 30]. As shown in these studies, adjustment for CTH is critical to achieve the reported high prediction accuracies.

Although many other studies have re-emphasized the critical need to adjust for cell-type heterogeneity when analysing DNAm data [31–33], it is surprising that many epigenome studies continue to ignore this major confounder when identifying biomarkers [34–37], or when proposing novel disease classifications [38–40]. In our opinion, there are two reasons for this. First, adjusting for cell-type heterogeneity, specially in complex solid tissues, can be technically challenging and investigators may not even be aware that tools for such adjustments or partial adjustments exist. For instance, all Cancer Genome Atlas (TCGA) projects [41–46] do not adjust for CTH when proposing novel cancer taxonomies. Second, there is a common belief that adjustment for cell-type heterogeneity is not really necessary when searching for biomarkers, based on the premise that any biomarker with high prediction accuracy is valuable irrespective of the underlying biological process driving the association. While this 2nd argument is entirely valid, it does not justify not performing additional analyses that adjust for cell-type heterogeneity (CTH), as these additional analyses can lead to important novel biological insight or novel biomarkers. The purpose of this perspective is therefore to reinforce the argument and rationale for performing adjustments for CTH, as well as to make the community aware that appropriate computational tools for such corrections are available and that they often work better and cheaper than laborious experimental alternatives (e.g. generating epigenetic profiles in purified samples).

## Why we should adjust for cell-type heterogeneity

**Fig. 1:**
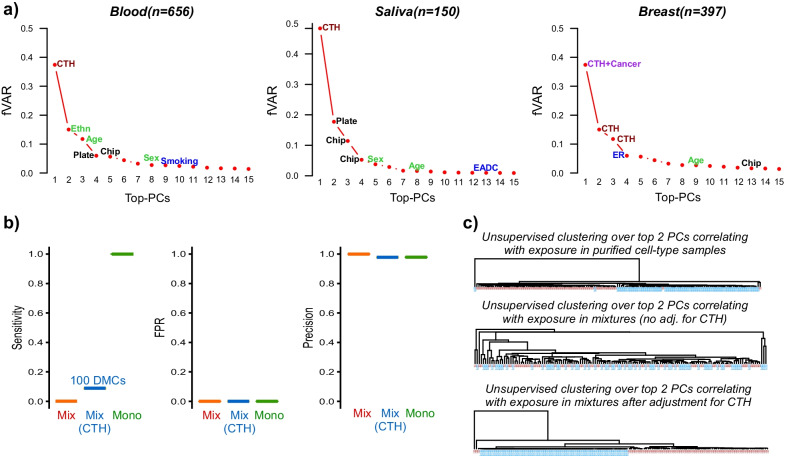
The need to adjust for CTH in epigenome studies. **a** A comparison of the relative data variance, expressed as a fraction of the total variance accounted by the top 15 PCs (*y*-axis, fVAR), explained by each of the top-15 principal components (PCs) (*x*-axis) for 3 separate epigenome studies, with datapoints annotated to the main factor driving that PC. CTH = cell-type heterogeneity; Ethn = ethnicity; EADC = esophageal adenoma carcinoma; ER = estrogen receptor status. The tissue-type and number of samples in each study are given above plots. These plots derive from Illumina DNA methylation data from the following published works: Blood [51], Saliva [49] and Breast [52]. Briefly, the blood dataset is from healthy individuals, saliva samples are from EADC patients and matched healthy controls, and the breast tissue data is from breast cancers and normal-adjacent tissue. In the case of blood, the top-PC correlates with CTH, PC-2 correlates with ethnicity and PC-3 with age. **b** Sensitivity, false positive rate (FPR) and precision to detect 1000 simulated DMCs introduced in 139 monocyte samples from BLUEPRINT with an exposure distinguishing 69 cases from 70 controls. In each panel, we display the metrics when inferring DMCs from realistic mixtures of 3 cell-types (neutrophils, CD4+ T cells and monocytes) (Mix, red), when inferring DMCs from these same mixtures whilst adjusting for CTH (Mix CTH, blue) and when inferring DMCs from the purified monocyte samples (Mono, green). **c** For the same simulated data as in (**b**), the unsupervised hierarchical clustering obtained when clustering the 139 monocyte samples over the top 2 PCs correlating with the exposure (top panel), when clustering the 139 mixtures over the top 2 PCs correlating with the exposure without any adjustment for CTH (middle panel), and when clustering the 139 mixtures over the top 2 PCs correlating with the exposure after adjustment for CTH (lower panel). Note that in the second case, i.e. when clustering over the top 2 PCs derived from the mixtures without adjustment for CTH, that these PCs only exhibited very marginal associations with the exposure, hence why the samples do not segregate by exposure

One important argument in favor of performing a differential DNAm analysis that adjusts for CTH comes from consideration of the relative data variance that can be attributed to the various factors, including CTH and the phenotype or exposure of interest. In general, given a genome-wide DNA methylation dataset where samples are drawn from different genetic backgrounds (e.g. ethnicity) and sexes, these factors are likely to dominate the data variance alongside CTH. That these three factors would dominate the DNAm variation landscape is intuitively clear. First of all, it is well known that a substantial proportion of DNAm is under genetic influence [47] and in many instances the effect sizes are quite substantial, i.e. over 50% DNAm differences between the homozygote A/A and B/B genotypes is common. Thus, top principal components (PCs) are likely to correlate with ethnicity if the study contains roughly equal numbers of samples from each ethnic group. Variation associated with sex is also expected to contribute substantially to the data variance, assuming that probes on the X and Y chromosomes are retained and assuming the study contains balanced numbers of each gender. In the case of CTH, a substantial proportion of the DNA methylome differs between major cell-types, e.g. between neutrophils and CD4+ T cells, or between epithelial and fibroblast cells [48–50], with over 80% differences in DNAm at individual loci being very common [49]. Thus, if cell-type composition of a tissue also varies between individuals, then this can be a major source of data variation, and indeed in the great majority of studies and irrespective of tissue-type, the top PC is most often driven by such variations in CTH (Fig. 1a). Depending on the phenotype or exposure of interest, the data variance driven by CTH could be substantially higher than that associated with the phenotype/exposure (Fig. 1a). For instance, components of variation associated with age or smoking are generally ranked much lower than those associated with CTH, with the corresponding variance often a factor of 5 or 10 lower than that associated with CTH. On the other hand, a phenotypic comparison between cancer and normal tissue is generally associated with a substantial remodelling of the DNA methylation landscape and would generally account for the top PC alongside CTH (Fig. 1a).

Hence, the importance of CTH adjustment is primarily a function of the relative data variance that can be attributed to the phenotype/exposure relative to CTH. For biomarker studies performed in easily accessible tissues like blood, serum, saliva, buccal swabs, vaginal swabs and cervical smears, the effect sizes associated with the typical phenotypes or exposures of interest are generally quite small (usually less than 20% DNAm changes), which means that the majority of the DNAm variation would be associated with CTH, clearly justifying the need to adjust for that variation.

A second important reason for performing the adjustment for CTH is that ultimately we would like to know if an observed DNAm change is independent of a change in cell-type composition, and if so, in which specific cell-types that alteration is happening in. This is clearly important if the aim is to understand how an exposure affects a specific cell-function, or which particular signaling pathways and cell-types are affected. The power to retrieve such important knowledge would be much reduced if DMCs were swamped by those arising due to changes in cell-type composition. In other words, the list of DMCs, and subsequent Gene Set Enrichment Analysis (GSEA) results would be much more informative if derived from a CTH-adjusted analysis.

To illustrate the negative impact that not adjusting for CTH can have, we performed a simple simulation experiment: using Illumina 450k DNAm data from BLUEPRINT encompassing 139 matched neutrophil, CD4+ T cell and monocyte samples [50], we artificially generated 1000 DMCs in 69 of the 139 purified monocyte samples, with effect sizes drawn randomly between 0.01 and 0.2 (i.e. 1–20% DNAm changes). Effect sizes of 1–10% are typical of EWAS done in blood and in relation to factors such as smoking [25], obesity [53] or age [54], whilst larger effect sizes (> 10%) are often observed in solid tissues in relation to a disease like cancer [55]. We here did not consider effect sizes larger than 20% because although such larger DNAm changes are indeed observed in relation to cancer [56] or genotype [57], they are generally speaking not as frequent as those involving smaller effect sizes. We further required the DMCs to be cell-type specific markers. We generated mixtures of the 3 cell-types using the 139 neutrophil and 139 CD4+ T cell samples matched to the 139 monocyte specimens, with cell-type fractions drawn from empirical distributions derived from whole blood [58]. We then computed the sensitivity, false positive rate (FPR) and precision when identifying DMCs from the mixtures, adjusting and not adjusting for CTH, and benchmarked these measures against those obtained by identifying DMCs from the purified monocyte samples themselves. This simple analysis clearly shows that not adjusting for CTH would lack power to detect any DMCs in a cell-type that is not a major component of the tissue (e.g. monocytes in whole blood) (Fig. 1b). In contrast, adjusting for CTH, the power would be 10% at a precision of over 95%, which means that we would be able to detect 100 DMCs with 95% confidence (Fig. 1b). Although in theory one could in principle build a highly accurate predictor from just one true DMC (a scenario of low sensitivity but high precision as captured by our simulation model), it is clear that the ability to detect a larger number of true DMCs is important for building more robust predictors. A clear real-world example where adjustment for CTH is critical to achieve high prediction accuracies (AUC > 0.8) has been in the context of diagnostic and pre-diagnostic cancer classifiers built from cell-free DNAm markers in serum [18, 19, 30]. In this context, the need to adjust for CTH stems from the fact that most cell-free DNA in serum derives from circulating lymphocytes, hence candidate biomarkers are often pre-screened by comparing DNAm patterns between cancer-tissue and blood [18, 19, 30].

The need for CTH-adjustment would also be important in the context of unsupervised classification analyses where the aim would be to propose novel molecular subtypes of a particular disease. In Fig. 1c we provide a clear example of how an unadjusted unsupervised clustering analysis would fail to detect hidden DNAm variation associated with an exposure or factor of interest. Indeed, it is worth mentioning here again that all major Cancer Genome Atlas (TCGA) projects [41–46] have proposed molecular classifications which are largely confounded by underlying variations in cell-type composition. Thus, most proposed molecular classifications of disease do not necessarily reflect the specific patterns of DNAm change present in the individual cell-types of a tissue. Instead, they predominantly reflect variations in cell-type composition. For instance, mesenchymal and immune-cell enriched subtypes have been observed in many different solid cancer types, including brain [59] and breast [41], and that these subtypes reflect increased presence of fibroblasts and immune-cells is now well established [60].

In relation to all previous arguments in favour of adjusting for CTH, we should clarify that this is always meant as an analysis to be done *in addition* to the standard unadjusted one. Indeed, an unadjusted analysis can detect shifts in cell-type composition that are associated with the phenotype or exposure of interest, and which could reflect important biological processes that have diagnostic or prognostic value. A case in point is the increased infiltration of CD8+ T cells in breast tumor tissue, which correlates with good outcome [61]. To emphasize this further, CTH-adjusted and unadjusted analyses largely yield complementary results and insights. For instance, by performing a CTH-adjusted analysis of buccal-swab DNAm profiles it has been shown that smoking-associated DNAm changes may differ between those occurring in the immune and squamous epithelial subsets of the tissue, with important implications for our understanding of how such DNAm alterations could mediate the risk of smoking-related diseases such as mouth cancer or cardiovascular disease [62, 63]. However, it is also worth pointing out that there could be other scenarios, for instance if the same DNAm changes are happening in all the underlying cell-types or in the dominant cell-type(s) of a tissue, where CTH-adjusted and unadjusted analyses would yield very similar results. For instance, there is evidence that DNAm changes associated with aging [54] and SNPs [50, 64] are largely independent of cell-type. In general, our proposed strategy to perform both adjusted and unadjusted analyses would lead to three classes of DMCs: (1) a set that is only significant in the unadjusted analysis, (2) a set that is only significant in the adjusted analysis, and (3) a set that is significant in both. As discussed above, their biological interpretations would be different: set (1) would correspond to biomarkers that are driven entirely by shifts in cell-type composition, set (2) would correspond to DMCs that are occurring in at least one of the cell-types and therefore independent of shifts in cell-type composition, whilst set (3) is more complex as it can include DMCs that are occurring in all underlying cell-types, or only in a predominant cell-type, or DMCs that are driven by both changes in cell-type composition as well as changes in individual cell-types. As to which set of biomarkers to take forward, the key priority would be to test their reproducibility using independent validation sets. Any biomarker that is highly reproducible and displays high sensitivity and specificity has great clinical potential, irrespective of whether it belongs to set (1), (2) or (3). Overall, our recommendation and guideline is to always perform both adjusted and unadjusted analyses, because only by doing both can we obtain a more complete picture and understanding of the observed DNAm changes.

## Adjusting for cell-type heterogeneity: feasibility

Most of the community is now well aware that adjustment for CTH can be accomplished with relative ease in tissues like blood or cord blood using what is known as a reference-based cell-type deconvolution algorithm [65, 66]. There are two elements necessary for a reference-based approach: (1) a DNAm reference matrix defined over a selected set of cell-type specific marker CpGs and all main cell-types within a tissue, (2) a statistical algorithm which, given this reference matrix and an independent DNAm profile of a sample, yields cell-type fraction estimates for all main cell-types in the given sample.

The main limitation of a reference-based approach is the availability or construction of a DNAm reference matrix. However, for specific tissues like blood and cord blood such DNAm reference matrices have been built [58, 65, 66] and adjustment for CTH in these tissues can be easily performed, at least at the resolution of 6–7 cell-types. It is worth highlighting here that the observed correlation between DNAm-based cell-type fraction estimates and those obtained experimentally (e.g. FACS/MACS-sorting) are excellent (typical *R*^2^ ~ 0.8), to the degree that the DNAm-based estimates could be viewed as providing the better gold-standard [67, 68]. For other tissues like saliva or buccal swabs, which contain squamous buccal epithelial cells in addition to the immune cells found in blood, reference-based cell-type deconvolution is possible using algorithms such as HEpiDISH [49], a method that infers cell-type fractions in a stepwise hierarchical fashion, first inferring fractions for the total epithelial and total immune cell fractions using an appropriately built DNAm reference matrix, and subsequently estimating fractions for all immune cell subsets using a separate carefully constructed DNAm reference matrix. These DNAm reference matrices are all available from the EpiDISH R-package and webserver [69]. For more complex solid tissues such as lung, which also contain stromal cells such as fibroblasts, the DNAm reference matrix within HEpiDISH can also be used to infer total epithelial, total fibroblast and total immune-cell fractions [49, 70]. A similar strategy is used by the MethylCIBERSORT algorithm [71]. More recently, the EpiSCORE algorithm [72], which leverages the high resolution nature of a tissue-specific scRNA-Seq atlas to impute a corresponding DNAm reference matrix, can be used to infer cell-type fractions in a much wider range of tissue types, including brain, liver, pancreas and skin. While it is clear that for solid tissue types the currently available DNAm reference matrices are not complete or may contain false positives, it is worth emphasizing that the inference of cell-type fractions is mathematically speaking a relatively robust procedure, i.e. it can tolerate missing minor cell-types or a number of false positives in the reference matrix [49]: similar to a voting algorithm, as long as the majority of the entries in the DNAm reference matrix are approximately correct, this will allow the algorithm to converge onto a relatively good proxy of the true cell-type fractions in the sample. Thus, while the quality of DNAm reference matrices for solid tissues will undoubtedly improve in the near future, either through improved imputation strategies, or because of improvements in single-cell methylomics that will enable direct construction of these reference DNAm matrices, the current ones are reasonably accurate to allow adjustment for CTH, as shown in the case of breast or lung tissue [72]. Indeed, CTH-adjustment when identifying DMCs should yield complementary and valuable insights to those of an unadjusted analysis. In particular, the ability to disentangle DMCs arising from a shift in cell-type composition from those present in the actual cell-types of the tissue is an important step towards a better understanding of the role of epigenetics in disease risk and disease progression. In addition, by estimating cell-type fractions in a given sample, this also opens up the possibility to infer cell-type specific differential DNAm using algorithms such as CellDMC [62], TOAST [73] and HIRE [74]. For instance, such an approach has led to the identification of a novel Endothelial-to-Mesenchymal transition (EndoMT) DNAm signature in lung cancer [72], or a novel smoking-associated DNA hypermethylation signature associated with acute myeloid leukemia [75].

## Conclusions

In summary, the scientific rationale for not adjusting for CTH when inferring biomarkers from complex tissues, or when proposing novel molecular classifications of disease is weak. Given that single-cell epigenomics will remain costly and unscalable to large numbers of individuals in the near future, computational methods offer a cheap and equally accurate means to adjust for CTH. We strongly recommend the use of such methodology for an improved and more complete interpretation of epigenomic and biomarker data.

## Data Availability

Not applicable.

## Abbreviations

DNAm: DNA methylation
scRNA-Seq: Single-cell RNA-seq
CTH: Cell-type heterogeneity
FFPE: Formalin-fixed paraffin embedded
TCGA: The cancer genome atlas
DMC: Differentially methylated cytosine
EWAS: Epigenome-wide association study
GSEA: Gene set enrichment analysis
FACS: Fluorescence-cytometric activated cell sorting
MACS: Magnetic activated cell sorting
